# Editorial: High-Throughput Sequencing-Based Investigation of Chronic Disease Markers and Mechanisms

**DOI:** 10.3389/fgene.2022.922206

**Published:** 2022-06-21

**Authors:** Yuriy L. Orlov, Wen-Lian Chen, Marina I. Sekacheva, Guoshuai Cai, Hua Li

**Affiliations:** ^1^ I. M. Sechenov First Moscow State Medical University (Sechenov University), Moscow, Russia; ^2^ Novosibirsk State University, Novosibirsk, Russia; ^3^ Institute of Cytology and Genetics SB RAS, Novosibirsk, Russia; ^4^ Agrarian and Technological Institute, Peoples’ Friendship University of Russia, Moscow, Russia; ^5^ Longhua Hospital, Shanghai University of Traditional Chinese Medicine, Shanghai, China; ^6^ Environmental Health Sciences, Arnold School of Public Health, University of South Carolina, Columbia, SC, United States; ^7^ Bio-ID Center, School of Biomedical Engineering, Shanghai Jiao Tong University, Shanghai, China

**Keywords:** high-throughput sequencing, biomarker development, chronic disease, disease mechanism, omics study, prognosis prediction

This “High-throughput sequencing-based investigation of chronic disease markers and mechanisms” issue focuses on the genomics studies on cancer and chronic diseases. With the recent development of sequencing technology and the rapid reduction of sequencing costs, high-throughput sequencing (including second and third-generation sequencing) is revolutionizing basic life science research and clinical research. High-throughput sequencing often produces millions of sequencing reads at a time, and the alignment or assembly of these reads allows determination of various mutations at the genomic level, accurate gene expression quantification at the transcriptomic level, and identification of histone or DNA modification at the epigenomic level. The resulting accumulation of enormous multi-omics information has opened up a new era of finding effective disease markers and studying their roles in disease occurrence and development (Anashkina et al., 2021).

Using high-throughput sequencing, various markers of chronic diseases have been developed at all omics levels, which have been used for diagnosis and classification of diseases, prediction of treatment effects, and prevention of diseases (Voronova et al., 2020; Glukhov et al., 2021). The chronic diseases include cancer, heart disease, diabetes, arthritis. The quickly and massively acquired multi-omics data, together with newly developed algorithms, provide excellent opportunities for the identification of more reliable biomarkers. This Research Topic aimed at 1) developing new chronic disease markers at four levels (i.e., genome, epigenome, transcriptome, and translatome) with the help of high-throughput sequencing, and 2) delineating potential marker-related mechanisms for chronic disease occurrence or development. More specifically, this Research Topic contains contributions including:

1) Identification of novel biomarkers and prediction signatures for chronic disease detection or prognosis prediction using high-throughput sequencing;
2) Analysis the possible pathological causes of markers as well as the potential roles they play in disease initiation and development;
3) Applications of new high-throughput sequencing techniques facilitating the development of more effective biomarkers of chronic disease;
4) New algorithms or tools for *in silico* identification of effective chronic disease markers based on high-throughput sequencing data.

Thus, we have organized this Research Topic to collect the papers focused on the frontiers of chronic disease markers. This Topic complements recent Research Topics “Bioinformatics of Genome Regulation” (Orlov et al., 2021a) and “Association between Individuals’ Genomic Ancestry and Variation in Disease Susceptibility” in *Frontiers in Genetics* (Das et al., 2022). The later journal issue collected papers focused on genetic background and ancestry rather than on molecular mechanisms of the human diseases (Das et al., 2022).

The papers published here extend the studies presented in the *Frontiers in Genetics* Topic “Bioinformatics of Genome Regulation (https://www.frontiersin.org/research-topics/17947/bioinformatics-of-genome-regulation-volume-ii), Volume II” and the earlier “Bioinformatics of Genome Regulation and Structure \ Systems Biology (BGRS\SB)” conference series (https://www.frontiersin.org/research-topics/8383/bioinformatics-of-genome-regulation-and-systems-biology; Orlov and Baranova, 2020; Orlov et al., 2016).

In this Research Topic a total of 14 papers could be arranged by two main areas—the cancer studies, and the works on the other chronic diseases such as allergy. Lung cancer is one of the leading causes of cancer-associated death in the world. We open the Research Topic by group of papers on lung cancer.

Non-small cell lung cancer (NSCLC) comprises about 85% of all lung cancers. Chang et al. used whole-exome sequencing to explore platinum-drug resistance mutations in advanced non-small cell lung cancer. Platinum-based chemotherapy is a fundamental treatment for non-small cell lung cancer (NSCLC) patients who are not suitable for targeted drug therapies. However, most patients progressed after a period of treatment. The authors enrolled nine NSCLC patients with platinum-based chemotherapy resistance, collected circulating tumor cells from them and performed whole-exome sequencing. Lu et al. studied response of TP53-negative NSCLC Patients to atezolizumab, an immune checkpoint inhibitor. This study provides a predictor, ubiquitin-like conjugation biological process gene mutation status, for identifying NSCLC patients who may have response to atezolizumab therapy.

Lung adenocarcinoma is the most common subtype of lung cancer with heterogeneous outcomes and diverse therapeutic responses. Wu et al. have developed novel risk-score model with eight miRNA signatures for overall survival of patients with lung adenocarcinoma. The authors selected eight microRNA (miRNA) signatures in The Cancer Genome Atlas. This model can also provide new insights into the current clinical staging system and can be regarded as an alternative system for patient stratification.

Wang et al. revealed the migration-associated genes involved in antitumor effects of herbal medicine Feiyanning on lung cancer cells using RNA-Seq and ATAC-Seq analysis. Traditional Chinese medicine formula Feiyanning has been clinically administered in China for more than a decade and raised attention due to its anticancer effect in lung cancer. Using cellular and molecular assays to examine the antitumor activities in lung cancer cells this study suggested that Feiyanning formula exerted the antitumor effects by modulating the expression and chromatin accessibility levels of migration-associated genes.

Breast cancer, the most commonly diagnosed cancer in women, has posed a major threat to women’s health globally. Hu et al. have compared pegylated liposomal doxorubicin (PLD) with epirubicin as adjuvant therapy for stage I-III breast cancer. Based on the large sample size and the long follow-up time of this study, the authors conclude that PLD has a similar anti-breast cancer efficacy as epirubicin while inducing lower level of cardiac toxicity in Han Chinese.

Gastric cancer is the third leading cause of cancer mortality across the world. Shi X. et al. studied CPSF6 protein in human gastric cancer. Alternative polyadenylation (APA) affects various biological functions and is involved in cancer. The authors found that 19 of 20 core APA factors were upregulated in gastric cancer tissues.

Esophageal cancer is the eighth most common cancer and the sixth leading cause of cancer death worldwide. Pang et al. estimated prognostic value of immune-related multi-IncRNA signatures associated with tumor microenvironment in esophageal cancer. The authors analyzed the tumor microenvironment for two subtypes of esophageal cancer, identified two multi-lncRNA signatures predictive for the prognosis, and explored the possibility of the signatures to forecast drug susceptibility.

The liver cancer ranks sixth in the number of new cases of malignant tumors worldwide and is the third leading cause of cancer death in the world. Hepatocellular carcinoma is the most common pathological type of primary liver cancer, accounting for about 90%. Shi H. et al. constructed a prognostic model based on the peptidyl prolyl cis–trans isomerase gene signature and explored it in patients with hepatocellular carcinoma.

Knyazev et al. studied hypoxia-related markers in inflammatory bowel disease. The authors detected expression activation of ITGA5 and PLAUR genes encoding integrin α5 and urokinase-type plasminogen activator receptor in inflammatory bowel disease specimens. The interaction of these molecules can activate cell migration and regenerative processes in the epithelium. These genes can serve as markers of inflammatory bowel disease progression and intestinal hypoxia.

The extracellular matrix (ECM) and cellular receptors constitute one of the crucial pathways involved in colorectal cancer progression and metastasis. Nersisyan et al. studied ECM–receptor regulatory network in colorectal cancer. The authors evaluated the prognostic information concentrated in the genes from ECM–receptor network using transcription factor and miRNA data and constructed two prognostic signatures.

Abnormal expression and regulation of non-coding RNA are involved in a variety of human diseases. Series of papers presenting miRNA and ncRNA studies focused on different groups of chronic diseases. Senile osteoporosis has recently become a major chronic metabolic bone disease in the world. Geng et al. aims to identify differentially expressed mRNAs and ncRNAs in senile osteoporosis patient-derived bone mesenchymal stem cells. By constructing the circRNA–miRNA–mRNA regulatory network, they found a ceRNA network (circRNA008876-miR-150-5p-mRNA) that could play an important role in senile osteoporosis.

The prevalence of allergic diseases has been increasing worldwide over the past 60 years, affecting about 30% of the global population. Qiao et al. described six male patients from unrelated families with a triad symptom of progressive postnatal slow growth, allergies, and fatty liver. The authors show association of imprinted gene SLC22A18 (solute carrier family 22 member 18) with this syndrome of variable allergy, short stature, and fatty liver. New variant in the promoter region of SLC22A18 is potentially associated with this syndrome.

Fei et al. studied potential associations and detect the underlying impact of single-nucleotide polymorphisms (SNPs) on proteinuria in kidney transplant recipients. The study suggested that the mutation of rs3804099 on the TLR2 gene was significantly related to the generation of proteinuria after kidney transplantation.

Wang et al. analyzed the expression and prognostic value of gamma-glutamyl-transferase 5 (GGT5) and its correlation with immune cell infiltration in gastric cancer. GGT5 may serve as a promising prognostic biomarker and a potential immunological therapeutic target for gastric cancer, since it is associated with immune cell infiltration in the tumor microenvironment.

Overall, we are proud of the Research Topic at *Frontiers in Genetics* we collated. We hope that the readers will find this collection stimulating and consider participation in upcoming conferences and journal issues in this area (https://bgrssb.icgbio.ru/2022/). Note also the thematic issue “Medical Genetics, Genomics and Bioinformatics” on the sequencing technologies applications in medical genetics (https://www.mdpi.com/journal/ijms/special_issues/Medical_Genetics_2022) and the continuing topic on gene expression mechanisms (https://www.frontiersin.org/researchtopics/17947/bioinformatics-of-genome-regulation-volume-ii) (Orlov et al., 2021a; 2021b). The complementary special issue at *Life* journal (https://www.mdpi.com/journal/life/special_issues/identification_HTS) continues collection of papers on the diseases markers and underlying molecular mechanisms (Snezhkina et al., 2021).
